# Evaluation of the Anatomical Locations of Stroke Events From Computed Tomography Scan Examinations in a Tertiary Facility in Ghana

**DOI:** 10.7759/cureus.14097

**Published:** 2021-03-25

**Authors:** Emmanuel K Edzie, Klenam Dzefi-Tettey, Philip Gorleku, Adu Tutu Amankwa, Ewurama Idun, Edmund K Brakohiapa, Obed Cudjoe, Frank Quarshie, Richard A Edzie, Abdul R Asemah

**Affiliations:** 1 Medical Imaging, University of Cape Coast School of Medical Sciences, Cape Coast, GHA; 2 Radiology, Korle-Bu Teaching Hospital, Accra, GHA; 3 Radiology, Komfo Anokye Teaching Hospital, Kumasi, GHA; 4 Radiology, 37 Military Hospital, Accra, GHA; 5 Radiology, University of Ghana Medical School, Accra, GHA; 6 Anatomical Sciences, University of Cape Coast School of Medical Sciences, Cape Coast, GHA; 7 Epidemiology and Public Health, African Institute for Mathematical Sciences (AIMS), Accra, GHA

**Keywords:** anatomical locations, stroke events, computed tomography scan, ghana

## Abstract

Introduction

Stroke events are leading causes of mortalities globally and currently increasing alarmingly in low- and middle-income nations including Ghana, thus overburdening national healthcare delivery sectors. This trend is predicted to ultimately have an impact on the socio-economic development of these countries, thus gaining the attention of policy-makers and implementers. This study was therefore conducted to evaluate the anatomical locations of stroke events from CT scan examinations and the possibly associated variables to assist in managing this non-communicable pandemic.

Methods

All computed tomography (CT) scans performed for stroke events at the Cape Coast Teaching Hospital from June 2016 to June 2020 were retrieved and reviewed for this study. The socio-demographics and the presence of hypertensive risk factor were also retrieved. Data were then collated, grouped, coded, inputted, and used for analysis. Chi-square test of independence was employed for assessing possible associations, and logistic regression analysis was performed to predict the anatomical locations of stroke events using sex and hypertension. Statistical significance level was specified at p ≤ 0.05.

Results

A total of 1,750 stroke cases were recorded during the study period, comprising 1,237 (70.7%) ischemic strokes and 513 (29.3%) hemorrhagic strokes. Majority (54.3%) of the patients were males. The average age of participants was 62.46±14.74 years. Basal ganglia (43.0%), parietal lobe (26.7%), and frontal lobe (6.9%) were the commonest anatomical locations. The elderly (≥ 60 years) were significantly affected at the basal ganglia (p=0.006), parietal lobe (p=0.005), frontal lobe (p=0.013), temporal lobe (p=0.048), and cerebellum (p=0.049). Basal ganglia lesions were significantly recorded in men, whereas lesions located at the pons were significantly seen in females. The regression model revealed that the risk of stroke at the pons increased by 2.155-folds in males (p=0.043; 95% CI=1.026-4.528). Generally, gender and hypertension were not significant predictors of stroke lesion locations.

Conclusions

The basal ganglia area, which falls under the middle cerebral artery territory, was the commonest anatomical location for stroke events in our setting. Knowing the anatomical locations of these stroke events has an impact on the type of interventions needed, especially at the early stages of these stroke events. CT perfusion, CT angiography, and magnetic resonance imaging (MRI) with MR angiography (MRA) when available can further assist in determining the exact cause so that urgent interventions such as endovascular treatments can be offered.

## Introduction

Stroke, or cerebrovascular accident (CVA), is a medical emergency that occurs as a result of a sudden onset of a focal neurological deficit or loss of neurologic function secondary to a vascular event persisting for more than 24 hours [1]. On the basis of etiology, strokes are categorized into two forms: ischemic or hemorrhagic strokes [2,3]. Around 80% of strokes are ischemic, which are characterized by thromboembolic cerebrovascular occlusions (blood clot in an artery in the brain). On the other hand, hemorrhagic stroke, which constitutes the remainder of strokes, is characterized by the rupture of blood vessels or aneurysm in the brain [2]. Stroke risk factors can be classified as nonmodifiable and modifiable. Some of the more commonly reported modifiable risk factors for stroke include hyperlipidemia, diabetes mellitus, hypertension, physical inactivity, and smoking. Sex, ethnicity, and age are the nonmodifiable risk factors [4].

Stroke is one of the leading causes of death in low- and middle-income nations and one of the top five leading causes of death in Ghana [5,6]. Although the actual prevalence rate of stoke in Ghana remains undetermined, the incidence rate and the death rate from stroke increase dramatically with age and accounted for approximately 9.1% and 13.2% of total medical adult admissions and deaths, respectively, between 2006 and 2007 [7]. Thus, stroke is a great medical and public health issue. As such, early and accurate diagnosis may significantly improve the morbidity and mortality rates.

With the invention of the computed tomography (CT) in the early 1970s, this imaging procedure has significantly facilitated the diagnosis and management of known and unknown conditions [8]. CT has since been used to diagnose and manage strokes and has added significantly to our understanding of pathophysiological brain alterations in humans [9].

CT is able to accurately diagnose and distinguish between ischemic stroke and hemorrhagic stroke. In addition, other conditions, such as subdural hematoma, that mimic stroke-like syndrome can easily be identified and differentiated by CT examination [10]. CT is one of the most accurate tools used for the recognition and localization of brain lesions. The acute and chronic phase that may develop following a sequence of stroke is clearly defined by CT [11]. Despite many improvements in magnetic resonance technology, CT still remains the primary imaging modality of choice for most of the patients and radiologists, and the most widely used for the evaluation of CVAs due to its fast acquisition of mages and its availability in most radiological practices in Ghana [12,13].

There have been several improvements in the diagnosis and management of strokes in recent years, providing significant information for stroke patients. As such, CT scan has been an important part of the evaluation and has established a more reliable framework for the control and use of intravenous contrast medium. The aims of this study are as follows:

I. To determine the anatomical locations of stroke events in a Ghanaian tertiary hospital.

II. To ascertain the associations between the socio-demographics of the population and the anatomical locations of strokes in our setting.

III. To determine which stroke type is likely to occur in which anatomical location.

IV. To find out the possible association of the anatomical locations of stroke events to hypertension, the commonest risk factor in our setting.

## Materials and methods

Study design, site, and participants

This retrospective cross-sectional study retrieved and reviewed all CT scans of the head performed for stroke to obtain the details of all the CT images and also the clinical details of all the patients diagnosed clinically with strokes. These patients were referred to the radiology unit in Cape Coast Teaching Hospital (CCTH) from June 2016 to June 2020. CCTH is the only public tertiary institution in south-central Ghana. It is the main referral center, providing a wide range of specialized tertiary services to the Central Region and its environs. CCTH is situated in Cape Coast, the capital city of the Central Region, along the Atlantic Ocean in the central part of southern Ghana. CCTH has a bed capacity of around 400 beds and is currently the training center for clinical students of the University of Cape Coast Schools of Allied Health Sciences and Medical Sciences.

CT scan image acquisition and interpretation

The brain images were obtained with a Toshiba Acquilion CT scanner, a multi-detector 16-slice CT (TSX-101A, Toshiba Medical Systems, Tochigi, Japan). A routine brain image acquisition protocol, starting from the lower most part of the skull (base) to the vertex, was used to acquire all the brain images using the following parameters: collimation of 1 x 16, tube current-exposure time of 225 mAs, rotation time of 0.75 s, tube voltage of 120 kV, and a slice thickness of 5 mm. A total of 1,750 stroke cases were retrieved and diagnosed over the study period. The acquired images over the study period were retrieved from the Picture Archiving Communication System (PACS) (IBM Watson Health Global Headquarters, Cambridge, MA, USA) and reviewed by two independent radiologists and two radiographers with over 10 years of neuroimaging experience. Disparities between reports of the head CT scans were resolved through discussion to attain a consensus.

Data collection

The diagnoses of the strokes made from all head CT scans over the study period irrespective of the type of stroke were included. The images were consecutively retrieved and evaluated from the PACS without any exclusions. Hypertension, which has been reported as the commonest risk factor in our setting and elsewhere, was also retrieved from the Lightwave Health Management Information System (LHMIS) by the researchers [14,15]. Hypertension was defined in this study as a sustained blood pressure elevation of (≥140 mm Hg systolic and ≥90 mm Hg diastolic) more than 72 hours after stroke onset [14]. The sex and age of the patients with stroke were also retrieved from the LHMIS. The anatomical locations of the stroke lesions were recorded and categorized under the following headings: basal ganglia, corona radiata, parafalcine, occipital, parietal, frontal, temporal, pons, cerebellum, and medulla.

Statistical analysis

The data obtained were collated, grouped, coded, inputted, and analyzed using Excel and Statistical Package for Social Sciences (SPSS) Version 21.0 (IBM Corp., Armonk, NY, USA) to estimate percentages and frequencies. Chi-square test was employed to check for possible association between the age, sex, type of stroke, and hypertension with the anatomical lesion locations. A logistic regression analysis was also performed to predict the anatomical locations of stroke using sex and hypertension, and the models that met goodness-of-fit test were presented. Statistical significance level was specified at p ≤ 0.05.

## Results

A total of 1,750 stroke cases were recorded during the study period, comprising 1,237 (70.7%) ischemic strokes and 513 (29.3%) hemorrhagic strokes. Majority (54.3%) of the patients in this study were males. The average age of participants was 62.46±14.74 years, with that of ischemic patients being significantly higher (63.11±13.73 years) than the hemorrhagic patients (60.89±16.83 years) (p=0.004). Among the stroke cases, the elderly group (≥60 years) had the highest recorded cases overall and also significantly dominated across the stroke types (p<0.001). Hypertension was significantly seen across the stroke types (p<0.001) (Table 1).

**Table 1:** Characteristics of patients Data are presented as n (%)

Item	Overall Count (%)	Type of Stroke		p-Value
		Ischemic	Hemorrhagic	
Total patients	1750	1237 (70.7%)	513 (29.3%)	-
Age				
Minimum	15	16	15	-
Maximum	106	99	106	-
Mean (SD)	62.46 (14.737)	63.11 (13.733)	60.89 (16.826)	0.004
Age group				
<18 years	2 (0.1%)	1 (50.0%)	1 (50.0%)	
18-39 years	117 (6.7%)	60 (51.3%)	57 (48.7%)	<0.001
40-59 years	540 (30.9%)	328 (70.7%)	158 (29.3%)	
≥60 years	1091 (62.3%)	794 (72.8%)	297 (27.2%)	
Gender				
Male	950 (54.3%)	668 (70.3%)	282 (29.7%)	0.711
Female	800 (45.7%)	569 (71.1%)	231 (28.9%)	
Hypertensive				
Yes	1054 (60.2%)	615 (58.3%)	439 (41.7%)	<0.001
No	696 (39.8%)	622 (89.4%)	74 (10.6%)	

**Figure 1:**
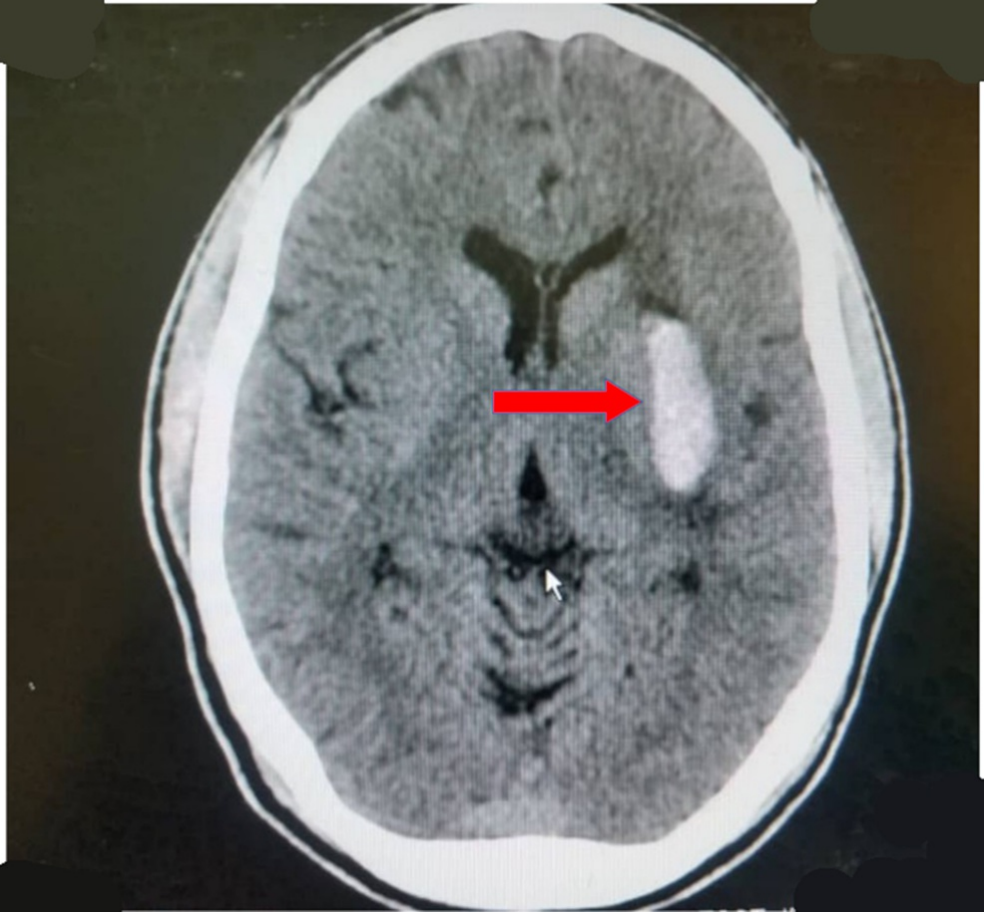
An axial non-enhanced CT scan of the brain of a 49-year-old woman, a known hypertensive who presented with a sudden onset of right hemiparesis, showing an area of hyperdensity at the left basal ganglia (red arrow) with minimal perilesional edema in keeping with acute left basal ganglia hemorrhage.

**Figure 2:**
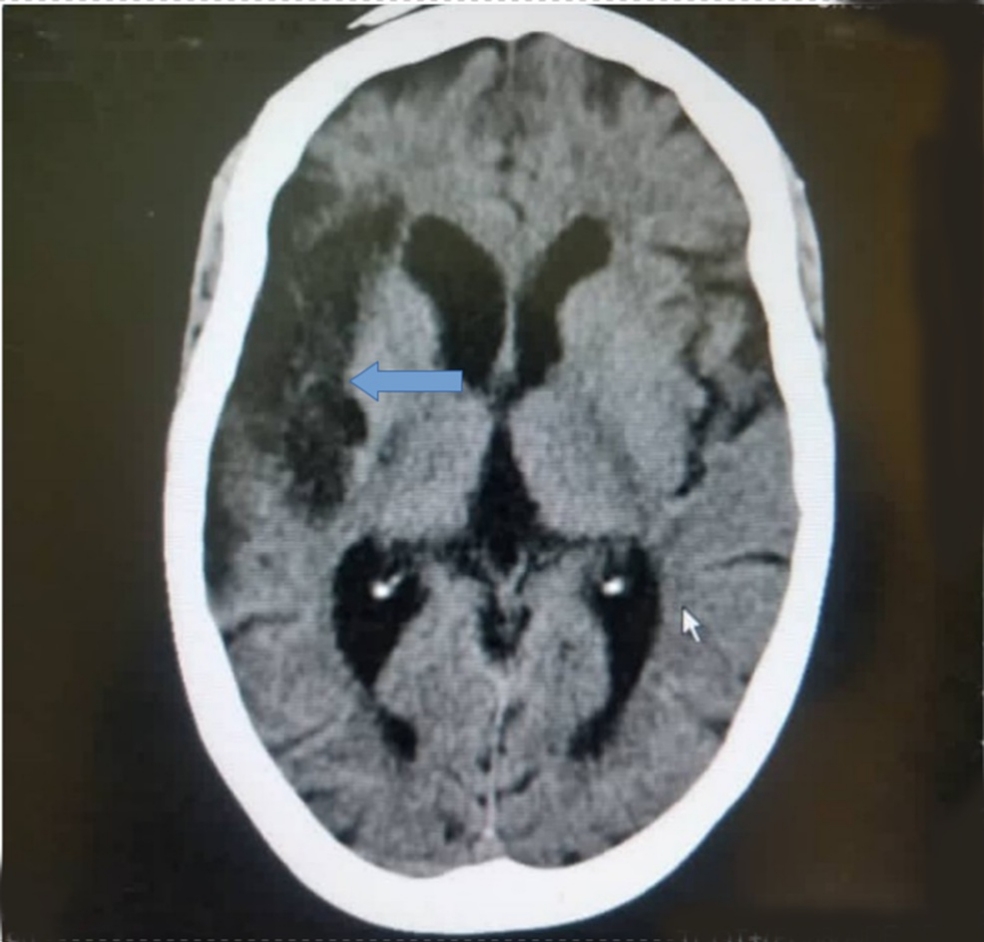
An axial non-enhanced CT scan of the brain of a 56-year-old man, hypertensive for 12 years, seen with a sudden onset of left hemiplegia and aphasia, showing an extensive area of hypodensity in the region of the right temporoparietal brain (blue arrow) with a comparative dilatation of the anterior horn of the ipsilateral lateral ventricle in keeping with right temporoparietal chronic infarct.

**Figure 3:**
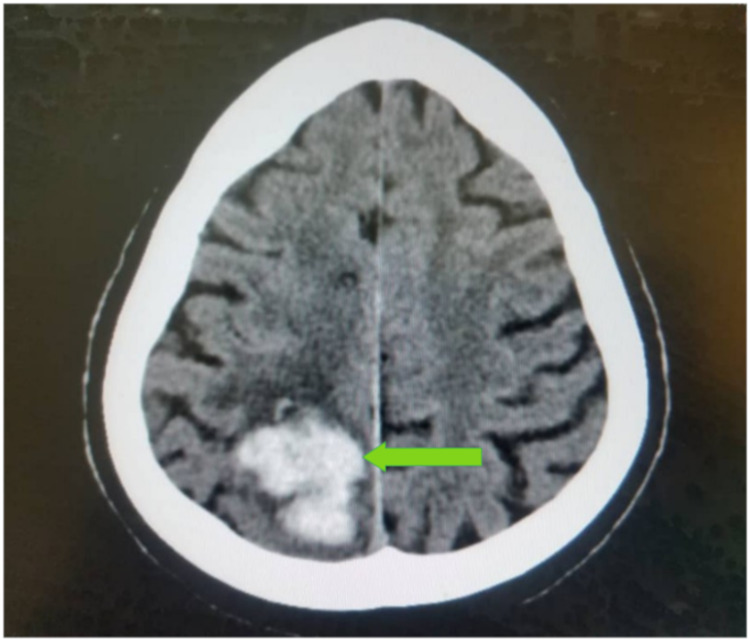
An axial non-enhanced CT scan of the brain of a 42-year-old man with a history of uncontrolled hypertension, who presented with a sudden onset of left-sided hemiplegia and blurred vision, demonstrating a hyperdense lesion in the right occipital lobe with acute blood attenuation with minimal perilesional edema (green arrow) in keeping with right occipital acute hemorrhage.

**Figure 4:**
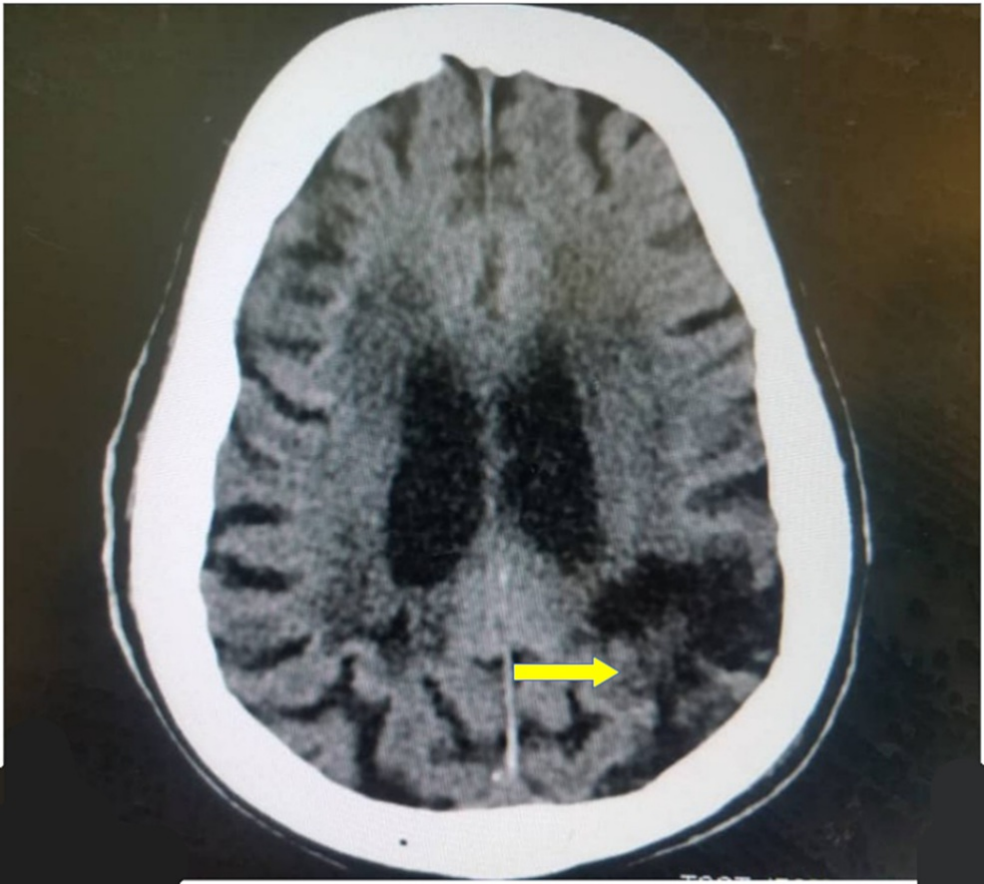
An axial non-enhanced CT scan of the brain of a 62-year-old female without a history of hypertension, who presented with a sudden onset of right-sided hemiplegia, showing a hypodense area at the left parieto-occipital region (yellow arrow) in keeping with intracerebral infarct (chronic).

Basal ganglia (43.0%) (Figure 1), parietal lobe (26.7%) (Figure 2), and frontal lobe (6.9%) stroke lesions were the most predominant locations noted on CT and were significantly seen in ischemic strokes (p=0.014, p<0.001, and p<0.001, respectively). Stroke lesions located at the occipital areas (Figures 3, 4) were significantly ischemic (p=0.020), whereas the pons was significantly affected by hemorrhagic strokes (p<0.001). The rest of the relationship between the anatomical locations of stroke events and stroke types is detailed in Table 2.

**Table 2:** Distribution of anatomical location of lesions from stroke events by types of stroke Data are presented as n (%)

Brain Lesion Location	Overall Count (%)	Type of Stroke		p-Value
		Ischemic	Hemorrhagic	
Basal ganglia	753 (43.0%)	509 (67.6%)	244 (32.4%)	0.014
Occipital lobe	116 (6.6%)	71 (61.2%)	45 (38.8%)	0.020
Parietal lobe	467 (26.7%)	389 (83.3%)	78 (16.7%)	<0.001
Frontal lobe	120 (6.9%)	66 (55.0%)	54 (45.0%)	<0.001
Temporal lobe	87 (5.0%)	61 (70.1%)	26 (29.9%)	0.905
Pons	31 (1.8%)	13 (41.9%)	18 (58.1%)	<0.001
Cerebellum	28 (1.6%)	17 (60.7%)	11 (39.3%)	0.243
Corona radiata	36 (2.1%)	26 (72.2%)	10 (27.8%)	0.838
Parafalcine	13 (0.7%)	7 (53.8%)	6 (46.2%)	0.199
Hemorrhage multiple locations	21 (1.2%)	-	21 (100.0%)	-
Infarct multiple locations	78 (4.5%)	78 (100.0%)	-	-

The elderly (≥60 years) were significantly affected by stroke lesions located at the basal ganglia (p=0.006), parietal lobe (p=0.005), frontal lobe (p=0.013), temporal lobe (p=0.048), and cerebellum (p=0.049). However, hemorrhagic strokes located at multiple sites were commonly noted in the younger age groups (<60 years) (p=0.018). Basal ganglia lesions were significantly recorded in men, whereas lesions located at the pons were significantly seen in females (Table 3).

**Table 3:** Distribution showing relationship between the socio-demographics and stroke lesion location Data are presented as n (%)

Brain Lesion Location	Age Group				p-Value
	<18 years	18-39 years	40-59 years	≥60 years	
Basal ganglia	0 (0.0%)	65 (8.6%)	214 (28.4%)	474 (62.9%)	0.006
Occipital lobe	1 (0.9%)	8 (6.9%)	31 (26.7%)	76 (65.5%)	0.294
Parietal lobe	0 (0.0%)	18 (3.9%)	162 (34.7%)	287 (61.5%)	0.005
Frontal lobe	0 (0.0%)	5 (4.2%)	24 (20.0%)	91 (75.8%)	0.013
Temporal lobe	0 (0.0%)	9 (10.3%)	36 (41.4%)	42 (48.3%)	0.048
Pons	0 (0.0%)	3 (9.7%)	15 (48.8%)	13 (41.9%)	0.138
Cerebellum	1 (3.6%)	4 (14.3%)	7 (25.0%)	16 (57.1%)	0.049
Corona radiata	0 (0.0%)	0 (0.0%)	13 (36.1%)	23 (63.9%)	0.153
Parafalcine	0 (0.0%)	0 (0.0%)	7 (53.8%)	6 (46.2%)	0.239
Hemorrhage multiple locations	0 (0.0%)	3 (14.3%)	12 (57.1%)	6 (28.6%)	0.018
Infarct multiple locations	0 (0.0%)	2 (2.6%)	19 (24.4%)	57 (73.1%)	0.138
Brain Lesion Location	Male		Female		p-Value
Basal ganglia	432 (57.4%)		321 (42.6%)		0.024
Occipital lobe	66 (56.9%)		50 (43.1%)		0.559
Parietal lobe	236 (50.5%)		231 (49.5%)		0.057
Frontal lobe	64 (53.3%)		56 (46.7%)		0.828
Temporal lobe	54 (62.1%)		33 (37.9%)		0.135
Pons	11 (35.5%)		20 (64.5%)		0.034
Cerebellum	13 (46.4%)		15 (53.6%)		0.400
Corona radiata	24 (66.7%)		12 (33.3%)		0.132
Parafalcine	7 (53.8%)		6 (46.2%)		0.975
Hemorrhage multiple locations	9 (42.9%)		12 (57.1%)		0.290
Infarct multiple locations	34 (43.6%)		44 (56.4%)		0.052

For the various anatomical locations of stroke events, hypertension was more commonly seen, but there was no significant association between hypertension and the various lesion locations, as demonstrated in Table 4.

**Table 4:** The relationship between lesion locations and hypertension Data are presented as n (%)

Brain Lesion Location	Hypertensive		p-Value
	Yes	No	
Basal ganglia	449 (59.6%)	304 (40.4%)	0.654
Occipital lobe	73 (62.9%)	43 (37.1%)	0.538
Parietal lobe	265 (56.7%)	202 (43.3%)	0.072
Frontal lobe	75 (62.5%)	45 (37.5%)	0.598
Temporal lobe	46 (52.9%)	41 (47.1%)	0.150
Pons	22 (71.0%)	9 (29.0%)	0.218
Cerebellum	19 (67.9%)	9 (32.1%)	0.406
Corona radiata	26 (72.2%)	10 (27.8%)	0.137
Parafalcine	9 (69.2%)	4 (30.8%)	0.506
Hemorrhage at multiple sites	17 (81.0%)	4 (19.0%)	0.154
Infarct at multiple sites	53 (67.9%)	25 (32.1%)	0.051

The regression model revealed that the risk of stroke at the pons increased by 2.155-folds in males (p=0.043; 95% CI=1.026-4.528). Generally, gender and hypertension were not significant predictors of stroke lesion locations, as shown in Table 5.

**Table 5:** Logistic regression analysis with gender and hypertension as the predictors of the various anatomical locations of stroke events/lesion

Item	p-Value	Odds Ratio	95% CI	
			Lower	Upper
Occipital lobe				
Gender	0.524	0.889	0.608	1.300
Hypertension	0.543	1.135	0.769	1.676
frontal lobe				
Gender	0.844	1.038	0.716	1.506
Hypertension	0.604	1.107	0.755	1.623
Temporal lobe				
Gender	0.151	0.722	0.463	1.126
Hypertension	0.169	0.738	0.479	1.137
Pons				
Gender	0.043	2.155	1.026	4.528
Hypertension	0.251	1.582	0.723	3.461
Cerebellum				
Gender	0.420	1.361	0.622	3.081
Hypertension	0.425	1.385	0.644	2.880
Corona radiata				
Gender	0.122	0.576	0.286	1.160
Hypertension	0.128	1.772	0.848	3.701
Parafalcine				
Gender	0.995	1.004	0.336	3.001
Hypertension	0.509	1.490	0.457	4.860

## Discussion

The advent of CT in neuroimaging has greatly improved the diagnosis of strokes by differentiating ischemic from hemorrhagic strokes, thereby paving the road for the necessary interventions to be employed [11]. This study found that ischemic strokes constituted majority (70.7%) of stroke cases (Table 1). Several studies globally have demonstrated similar trends in the proportions of stroke types [16-19]. Our study also revealed that males had the majority of strokes overall and more dominant across the various types of strokes. This finding is in agreement with previous studies that have reported a higher prevalence of stroke events in men compared to women [18-20]. A study assessing stroke incidence by sex across the lifespan found that women have a lower hazard of stroke compared to men [21]. The actual reason for this trend cannot be readily explained in this current study.

Studies in Ghana by Agyemang et al. and Edzie et al. have found the mean age of stroke events to be between 62 and 64 years [7,13]. These findings are comparable to the mean age of 62.46 years found in this study (Table 1). The age of onset of stroke in Africa is much lower and estimated to be between 55 and 65 years, whereas that of the developed countries have been noted to be much higher (>70-75 years) [22]. This emphasizes that the burden of stroke in African occurs in the younger generation compared to the developed countries. This is likely to have a dire impact on productivity in an already overstretched lower income economies. Majority of the stroke cases in this study were seen in the elderly group (>60 years) (Table 1). A reason for this may be the fact that the risk of stroke increases with increasing age and that the incidence doubles with each decade of life after the age of 45 years [23].

Analysis of data from CT scan for this current study showed that the commonest anatomical locations for stroke events were the basal ganglia (Figure 1) followed by the parietal lobe (Figure 2) and then the frontal lobe, and patients with these events also significantly suffered more ischemic strokes than hemorrhagic strokes (p=0.014, p<0.001, and p<0.001, respectively) (Table 2). Our findings differed from those published in a study conducted in Nigeria by Kolade‑Yunusa et al. who reported the parietal lobe to be the commonest stroke location followed by the frontal lobe [20]. Although CT perfusion and angiography were not undertaken in our study due to its unavailability in our setting, the main cerebral arterial territories corresponding to the common locations found in this study were the middle cerebral arteries (MCA) and anterior cerebral arteries. A study in South Africa by Daffue and Joubert assessing CT stroke findings also reported the MCA as the commonest vascular territory for strokes, making the Ghanaian population similar to other studied population [24]. The basal ganglia, parietal lobe, frontal lobe, temporal lobe, cerebellar, and hemorrhagic strokes located at multiple sites were significantly seen in this study to be increasing with age (Table 3). Males were more commonly affected at the basal ganglia, whereas females showed predilection toward the pons (Table 3).

Our study revealed that majority (60.2%) of the patients in this population had hypertension, and subsequently hypertension was observed to be present for the majority of stroke events in all the anatomical locations (Table 4). Mensah et al. reported that, in Africa, hypertension is recorded in more than 90% in hemorrhagic stroke patients, whereas more than 50% of ischemic stroke patients had high blood pressure; thus, it is not surprising to find it be more common in our patients [25]. Even though previous studies have reported hypertension as a common risk factor for stroke irrespective of stroke location, the current study found no significant association between hypertension and the anatomical locations of stroke events [15,25].

Limitation of the study

Very early or minor strokes could have been missed in this study because of the current unavailability of CT angiography and perfusion in our setting. Some locations could have been missed from stroke events, which resulted in deaths prior to imaging. Only hypertensive risk factor was considered for this study, which is a limitation. The relatively smaller number of stroke events considered in this study is another limitation. The continual acquisition of imaging equipment with higher specifications in Ghana will warrant future investigations with advanced CT software such CT perfusion, CT angiography, and MRI in order to overcome some of the aforementioned limitations

## Conclusions

The basal ganglia area, which falls under the MCA territory, was the commonest anatomical location for stroke events in our setting followed by the parietal lobe and then the frontal lobe. Knowing the anatomical locations of these stroke events has an impact on the type of management needed, especially at the early stages of these events. CT perfusion, CT angiography, MRI, and MR angiography, when available, can further assist in determining the exact cause for urgent interventions. Men significantly suffered more stroke events at the basal ganglia, whereas strokes located at the pons were more commonly seen in females. Ischemic strokes were more common in all anatomical locations for stroke events except for the pons, which showed significantly hemorrhagic strokes.
